# Nanopore Size-Dependent Raman Spectroscopy of Two-Dimensional γ‑Graphyne

**DOI:** 10.1021/acsomega.5c05720

**Published:** 2025-12-13

**Authors:** João Marcelo de Almeida Garcia, Dattatray Jaysing Late, Marcos Assunção Pimenta, Jenaina Ribeiro-Soares, Raphael Longuinhos Monteiro Lobato

**Affiliations:** † Departamento de Física, 67739 Universidade Federal de Lavras, Lavras, Minas Gerais 37200-000, Brazil; ‡ Departamento de Física, 28115 Universidade Federal de Minas Gerais, Belo Horizonte, Minas Gerais 30270-901, Brazil; § Departamento de Física, Universidade Federal de Ouro Preto, Ouro Preto, Minas Gerais 35400-000, Brazil

## Abstract

Graphynes are allotropes of carbon that display both *sp* and *sp* ^2^ carbons, which form acetylene- and benzene-like units, respectively, connected in a single-atomic layer structure. The *sp–sp* ^2^ networks display rich structural patterns, with different physical and chemical properties, promising for clean energy generation and storage, optoelectronics, heat dissipation, and molecular filter applications. Here, we apply density-functional theory calculations to simulate the structure and Raman spectrum of γ-graphyne monolayers with one to six acetylene units between its carbon rings. Ready-to-use general formulas relating the lattice parameter, nanopore size, and number of Raman-active lattice vibrations to *N* are given. The wavenumber dependence of the G, G′, Y, and Y′ modes on *N* reveals differences of the order of tens of cm^–1^. These results provide tools for fast and reliable identification of the *N*-type γ-graphynes, which is key for the architecture design of molecular filters and other nanodevices.

## Graphical abstract

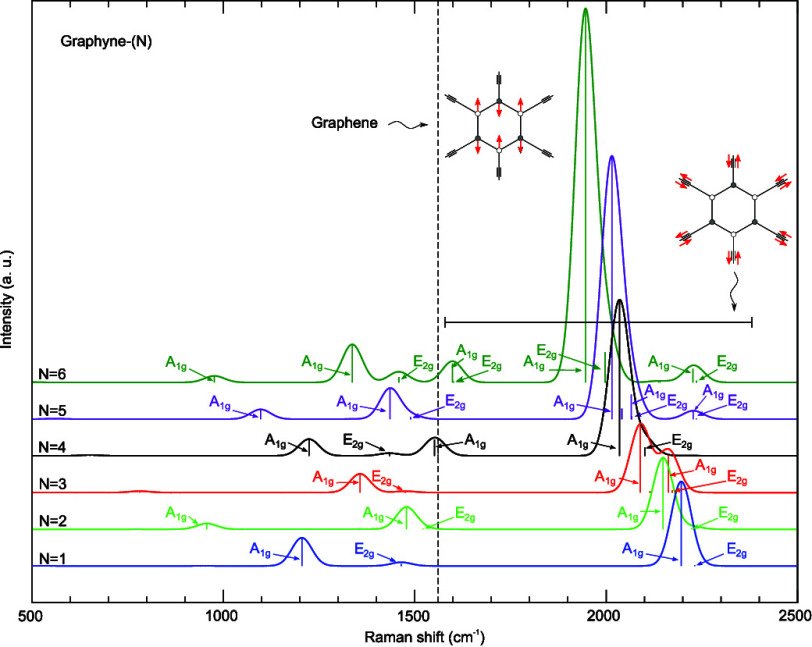

## Introduction

The richness of carbon atomic orbital hybridization leads to the largest family of allotropes. The most well-known allotropes of carbon have their atoms in *sp* ^3^- (diamond), *sp* ^3^- and *sp* ^2^- (amorphous carbon), and *sp* ^2^-hybridization states (graphite/graphene, carbon nanotubes, and fullerenes). Less well-known family members include lonsdaleite, where the carbon atoms are in *sp* ^3^-like hybridization, forming a hexagonal diamond structure,^1–4^ and carbon atom wires, where the carbon atoms are in *sp*-hybridization.^5–10^

Graphynes are new allotropes of carbon with *sp* and *sp* ^2^ carbons, forming *sp*–*sp* ^2^ single-atom-layer networks, which were predicted in the 1980s.^11^ Although metastable with respect to graphite, they display high thermal stability, with lifetimes of free-standing single-layer graphynes estimated to be more than 10^44^ years at room temperature and conversion to graphene occurring only at temperatures above 2000 K.^12^ They can be “obtained” by inserting acetylene-like units (−C≡C−)_*n*_ between the *sp* ^2^ bonds in graphene^13,14^ in different proportions, resulting in a variety of materials: α- (in each *sp* ^2^ bond), β- (in two out of three *sp* ^2^ bonds), γ- (in one out of three *sp* ^2^ bonds), and 6,6,12-graphynes (in five out of 12 *sp* ^2^ bonds).^11,15–17^ They display promising chemical, mechanical, electrical, thermal, and optical properties for various applications, starting to be realized after their recent experimental synthesis.^17–27^

The γ-graphyne with (−C≡C−)_*n*_ in one out of three *sp* ^2^ bonds in graphene, or graphyne-(*N*) from now on, can be viewed as a network of carbyne-like chains connecting aromatic rings (Figure 1).

**Figure 1:**
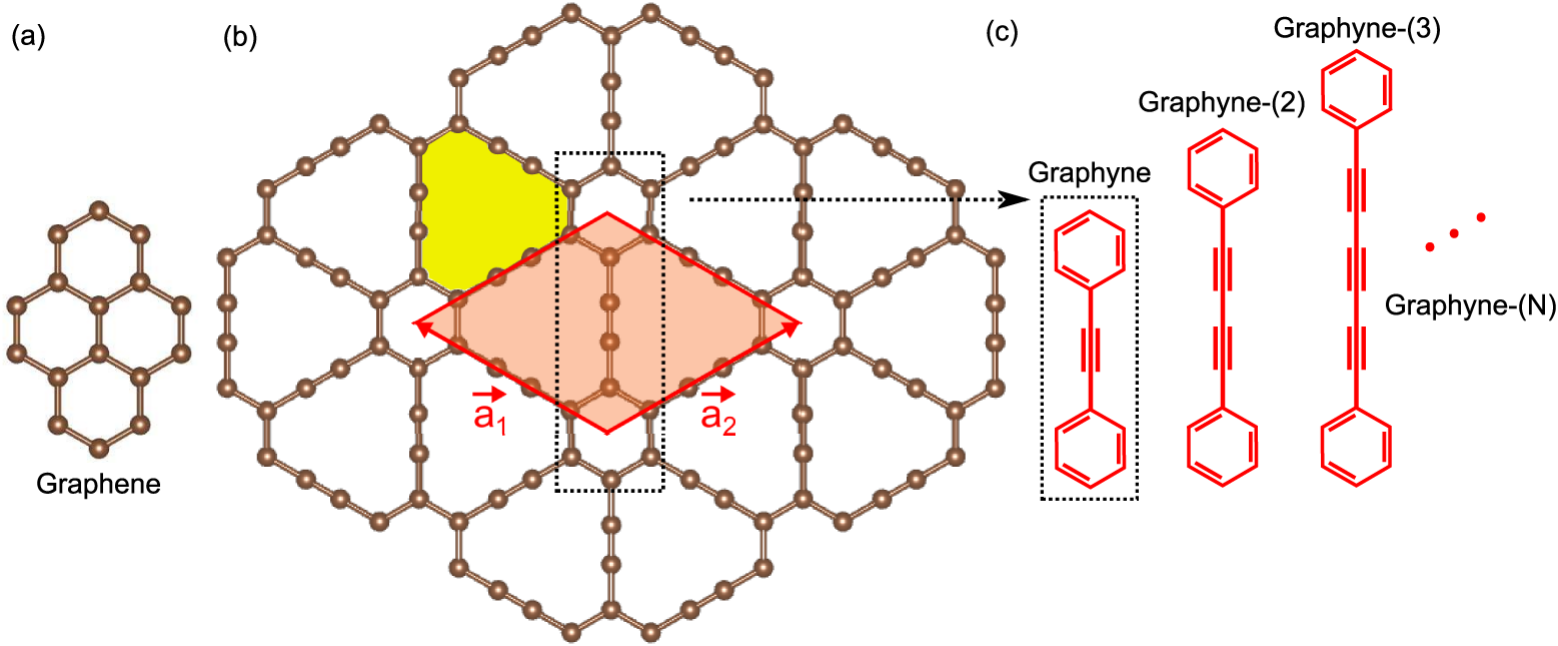
Atomistic model of (a) graphene and (b) graphyne or graphyne-(1). The graphyne-(1) unit cell (and primitive vectors) and its nanopore are colored red and yellow, respectively. (c) Illustration of increasing the number *N* of acetylene-like units between the aromatic rings, giving the origin to graphyne-(*N*).

Graphynes-(*N*) are promising for applications in water desalination. The process of separating salt ions and water permeability was simulated for the cases of graphyne-(3) and graphyne-(4), presenting relevant results.^28,29^ In addition, ref 30 shows that graphyne membranes are able to reject salts of divalent heavy metals (e.g., copper sulfate) and monovalent inorganic salts (e.g., sodium chloride). Graphyne-(2) (graphdiyne)^31^ and Graphyne-(4) (graphtetrayne)^24,32^ were synthesized and have semiconducting properties, the former displaying conductivity comparable to silicon^31^ and has also been used as an anode in lithium batteries.^33^ In addition, graphyne-(2) nanotubes were also realized in the laboratory.^34^ These results have renewed effort toward graphyne synthesis and applications.

Variations in the number of layers or atomic coordination result in different numbers and symmetries of lattice vibrational modes in layered materials.^35–39^ Raman spectroscopy is especially suitable for probing the physical and chemical properties of carbon allotropes.^40^ The Raman spectra of graphynes with a single acetylene-like unit (α-, β-, and γ-) were simulated,^8,18,41,42^ and experimental studies in the case of graphyne-(2) and graphyne-(4) were performed,^31,32,43^ showing great potential to distinguish their structures.

In this work, first-principles calculations are used to simulate the geometry and the Raman spectrum of six variations of γ-graphyne, with one to six acetylene-like units, allowing us to unveil the *N*-dependence of these properties.

## Computational Methods

The geometric relaxation and Raman spectrum calculations were done via density-functional theory (DFT)^44,45^ and density-functional perturbation theory (DFPT)^46,47^ with norm-conserving pseudopotentials,^48,49^ using the QUANTUM-ESPRESSO distribution.^50^ The electronic exchange-correlation interaction was approximated by the local-density approximation (LDA) functional,^51^ which provides excellent estimations for the structural parameters and phonon wavenumbers in insulators,^52,53^ semiconductors,^39,41,54,55^ and metals.^56,57^ For graphyne-(1), we sampled the Brillouin zone with an 8 × 8 × 1 grid.^58^ To keep the distance between points in the k-space similar to the values in graphyne-(1), we used an 8 × 8 × 1 grid for graphyne-(2), a 6 × 6 × 1 grid for graphyne-(3), a 5 × 5 × 1 grid for graphyne-(4), and 4 × 4 × 1 grids for graphyne-(5) and graphyne-(6). The vacuum between the images was set to 15 Å. The geometries were optimized until forces and stress were lower than 0.1 m*E*_h_ Å^–1^ and 50 MPa, respectively. The plane wave kinetic energy cutoff was set to 30 Ha. These parameters result in values for the phonon wavenumbers in graphyne-(1) and graphyne-(2) that differ by up to 1% from those found in Zhang et al.^41^

The selection rules were derived following Ribeiro-Soares et al.^37,38^

## Results and Discussion

### Structure

The γ-graphyne family, or graphyne-(*N*), is two-dimensional structures with hexagonal symmetry, which belong to the centrosymmetric space group *P*6/*mmm* (#191 space group,^59^ or $D_{6h}^{1}$ according to the Schönflies notation); the same observed in graphene (see Figure 1).

Graphynes-(*N*) can be obtained by “inserting” acetylene-like units (*sp* carbons), forming carbyne chains, and bonding carbon hexagons (*sp* ^2^ carbons). The structure of graphyne-(1) is shown in Figure 1b, and the cases of graphyne-(1 to 3) are illustrated in Figure 1c. The unit cell and primitive vectors of graphyne-(1) are also represented in Figure 1 and can be defined similarly for other graphynes-(*N*).

Figure 2 presents the structural parameters of the graphyne-(1 to 6).

**Figure 2:**
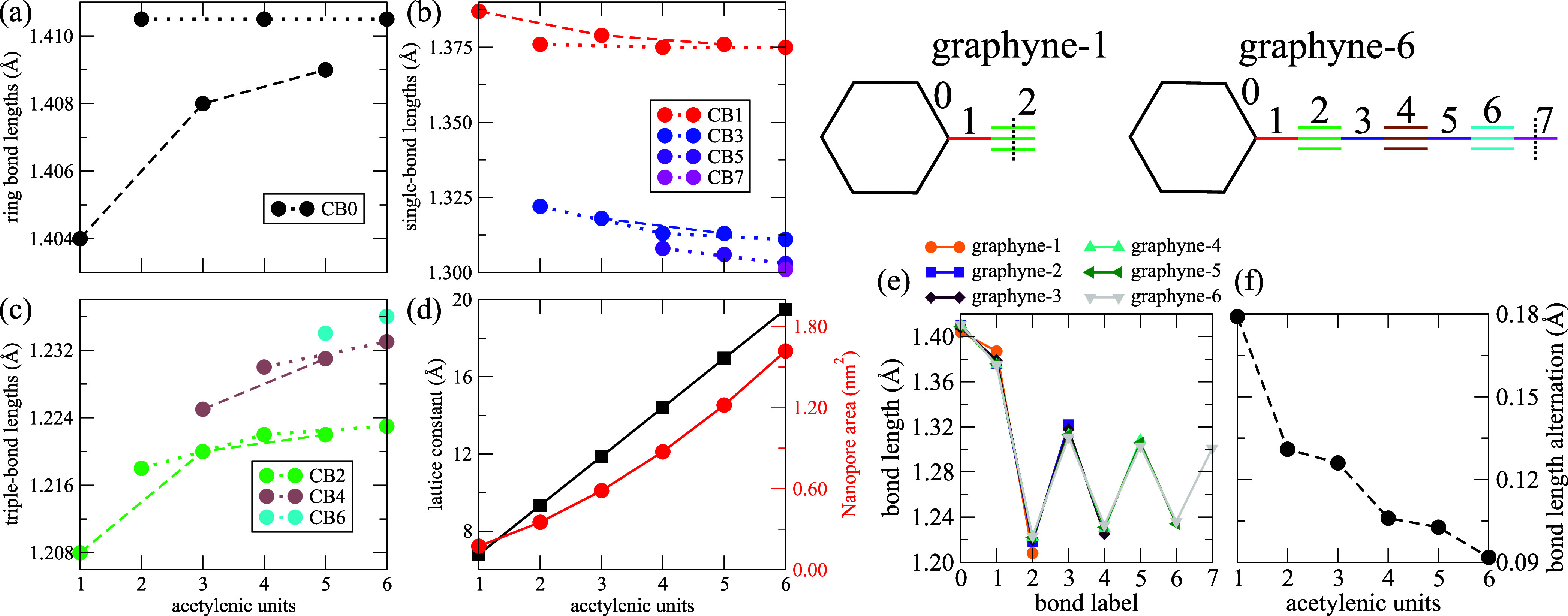
Dependence of graphyne-(*N*) structural parameters on the number *N* of acetylene-like units. At the upper right, we define the labels of the nonequivalent carbon bonds (CB), showing the cases of graphyne-(1) and graphyne-(6). The dashed line perpendicular to the CB defines the mirror plane of the carbyne chain. (a) Bond lengths in the carbon ring (CB0). (b) Bond lengths of the link between the carbon ring and carbyne chain (CB1) and the single bonds in the carbyne chain (CB3, CB5, and CB7). (c) Bond lengths of the triple bonds in the carbyne chain (CB2, CB4, and CB6). The dotted and dashed lines follow the evolution of the specified bonds when the number of acetylenic units is *N*-even and *N*-odd, respectively. We do not connect CB6 and CB7 because we consider only graphyne-(1 to 6). (d) Lattice constant (left *y*-axis) and nanopore area (right *y*-axis). The solid line displays fitted curves. (e) Unified view of the bond lengths of the CB, where the *x*-axis is the bond label. (f) Bond length alternation (BLA).

The nonequivalent carbon bonds (CBs) are labeled and colored as in the drawings of graphyne-(1) and graphyne-(6), shown at the upper right of Figure 2. The dashed line perpendicular to the bonds represents the mirror plane of the carbyne chain. These CBs are in the carbon ring (CB0), the terminal carbon bond between the carbon ring and the carbyne chain (CB1), and the alternating triple bonds (CB2, CB4, CB6) and single bonds (CB3, CB5, CB7) in the carbyne chains. In Figure 2a–c the dotted and dashed lines follow the CBs for *N*-even and *N*-odd numbers of acetylenic units, respectively. CB6 occurs in graphyne-(5) and graphyne-(6); thus, the circles representing these values are not connected. Also, CB7 occurs only in graphyne-(6) and is represented by an isolated circle.

The CB0 bond lengths in the carbon ring in graphyne-(*N*) have the same value, *l*_CB0_, constrained by symmetry. Their lengths are listed in Figure 2a. The *l*_CB0_ values show different trends for *N*-even and *N*-odd. In the case of *N*-even, the *l*_CB0_ values are essentially independent of *N*, while in the *N*-odd case, they increase monotonically with *N* toward the value of the *N*-even case.

Figure 2b presents the lengths of the single bonds in graphyne-(*N*). The CB1 bond lengths form a separate group from the single bonds within the carbyne chain (CB3, CB5, and CB7). The *l*_CB1_ values of the *N*-even case are also essentially independent of *N*. The *l*_CB1_ of *N*-odd case decrease with N, converging to the value in the *N*-even case. The bond lengths of the single bonds within the carbyne chain decrease with *N*; each new single bond that arises with increasing *N* begins smaller than those already present, e.g., *l*_CB3_ > *l*_CB5_ > *l*_CB7_ for all *N* values.

Figure 2c shows the triple bond lengths for graphyne-(1 to 6). The bond lengths of the triple bonds within the carbyne chain increase with *N*; each new triple bond that arises with increasing *N* begins larger than those already present, e.g., *l*_CB2_ < *l*_CB4_ < *l*_CB6_ for all *N* values. Also, the bond lengths of the triple bonds in the *N*-even cases increase less with *N* than in the *N*-odd cases, and the latter seem to converge to the values of the former.

The left *y*-axis of Figure 2d shows the *N*-evolution of the unit cell length (lattice parameter), which equals the distance between the centers of the adjacent aromatic rings. The unit cell length increases with an increase in the number of acetylene-like units (*N*). These trends were also found in previous theoretical studies of the lattice constants of graphyne-(1 to 4).^41,60^ By curve-fitting our results, we estimate the unit cell length of graphyne-(*N*) to be given by

1 f(N)=4.252⁡Å+N2.539⁡Å

The right *y*-axis of Figure 2d displays the area between the aromatic rings in graphyne-(*N*), i.e., nanopore size, for graphyne-(1 to 6). The curve-fitting of these values provides the trend for the nanopore size in graphyne-(*N*):

2 $$f\left(N\right)=0.052{\mathrm{nm}}^{2}+N0.094{\mathrm{nm}}^{2}+N^{2}0.028{\mathrm{nm}}^{2}$$

Graphyne-(*N*) are promising molecular filters due to these nanopores (see Figure 1b), and their sizes are key for filter selectivity.^28–30^

Figure 2e presents the *l*_CB_ value in a unified view, where the *x*-axis is the bond label of the CB. Figure 2f displays the bond length alternation (BLA) dependence on the number of acetylenic units in the carbyne chain. The reduction of the BLA with the increase of the carbyne chain is attributed to the increase in π-conjugation.^6,7^

### Raman-Active Phonons in γ-Graphynes

As the number *N* of acetylene-like units increases, the number of atoms in the unit cell increases as 6­(*N* + 1), raising the number of phonon modes. Among the silent, infrared- and Raman-active modes in graphynes-(*N*), we focus on the latter group. Considering the structural details, group-theory analysis reveals that the expected γ-graphynes-(*N*) Raman-active modes equal:

3 $$\Gamma =\left(N+1\right)\left(A_{1g}⊕E_{1g}⊕2E_{2g}\right)$$

This formula contains the cases of graphyne-(1),^18,41,61^ graphyne-(2),^41^ as well as that for an arbitrary number of acetylene-like units (−C≡C−)_*n*_ introduced as carbyne chains between the aromatic rings.

The Raman scattering intensity of the graphyne-(*N*) Raman-active modes^62^ is given by $I\propto {|{ê}_{s}\cdot \mathrm{R⃡}t\cdot {ê}_{i}|}^{2}$ , with *ê_s_* and *ê_i_* representing the incident and scattered radiation polarization unitary vectors, respectively, and *R⃡t* is the Raman tensor for a given vibrational mode. The Raman tensors for the modes expected in γ-graphynes are^62^

$$A_{1g}:\left(\begin{array}{ccc} a & 0 & 0\\ 0 & a & 0\\ 0 & 0 & b \end{array}\right)$$

$$E_{1g}:\left(\begin{array}{ccc} 0 & 0 & 0\\ 0 & 0 & c\\ 0 & c & 0 \end{array}\right),\left(\begin{array}{ccc} 0 & 0 & -c\\ 0 & 0 & 0\\ -c & 0 & 0 \end{array}\right)$$

$$E_{2g}:\left(\begin{array}{ccc} d & 0 & 0\\ 0 & -d & 0\\ 0 & 0 & 0 \end{array}\right),\left(\begin{array}{ccc} 0 & d & 0\\ d & 0 & 0\\ 0 & 0 & 0 \end{array}\right)$$

In backscattering setup, the *ê_i_* and *ê_s_* can then be defined as |1, 0, 0⟩ and |cos­(θ), sin­(θ), 0⟩, respectively, resulting in *I*(*A*_1*g*_) ∝ *a* ^2^ cos^2^(θ), *I*(*E*_1*g*_) ∝ 0, and *I*(*E*_2*g*_) ∝ *d* ^2^. From these results, the *E*_1*g*_ modes are not expected to be observed in this geometry, while polarization-dependent measurements for the scattered light will result in a dumbbell pattern for *A*_1*g*_ modes and a circular pattern for *E*_2*g*_ modes. These results indicate the inclusion of polarizers in parallel and cross configurations to distinguish the *A*_1*g*_ from the *E*_2*g*_ modes; i.e., the former is not observed in the cross configuration, and the latter is observed in both cases.

Figure 3 shows the calculated Raman spectra of powder graphyne-(1 to 6), i.e., a powder of monolayers randomly oriented, where the *A*_1*g*_ and *E*_2*g*_ modes with the highest relative scattering intensity are indicated; these are the modes with the highest probability to be observed experimentally. The figure also depicts two representative graphyne-(1) vibration patterns: the C–C stretching in the aromatic ring (this band is degenerate, and we show only one of the vibration patterns; the other is orthogonal to it) and the vibration of the acetylenic units (there are degenerate and nondegenerate bands in these cases, and our illustration is only to highlight that the aromatic ring is motionless). The vertical dashed line represents the wavenumber related to the C–C stretching in the aromatic ring in graphene, here calculated and equal to 1561.3 cm^–1^.

**Figure 3:**
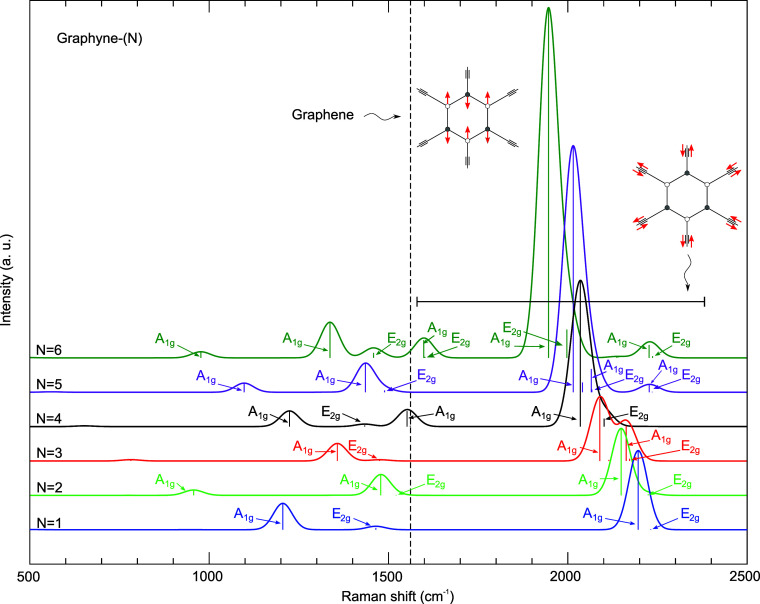
Raman spectra of powder graphynes-(*N*). The intensity of each spectrum is normalized to its most intense scattering peak. The spectra were stacked and smoothed (Gaussian integration) without physical meaning. Representative graphyne-(1) vibration patterns are illustrated. The vertical dashed line indicates the graphene *E*_2*g*_ wavenumber.

The Raman shifts in powder graphyne-(1 to 6) are given in Table 1, with their respective assignments and nonresonant scattering intensities, normalized by the highest value for each graphyne-(*N*). This table provides details of the Raman-active mode structure not seen in Figure 3.

**Table 1:**
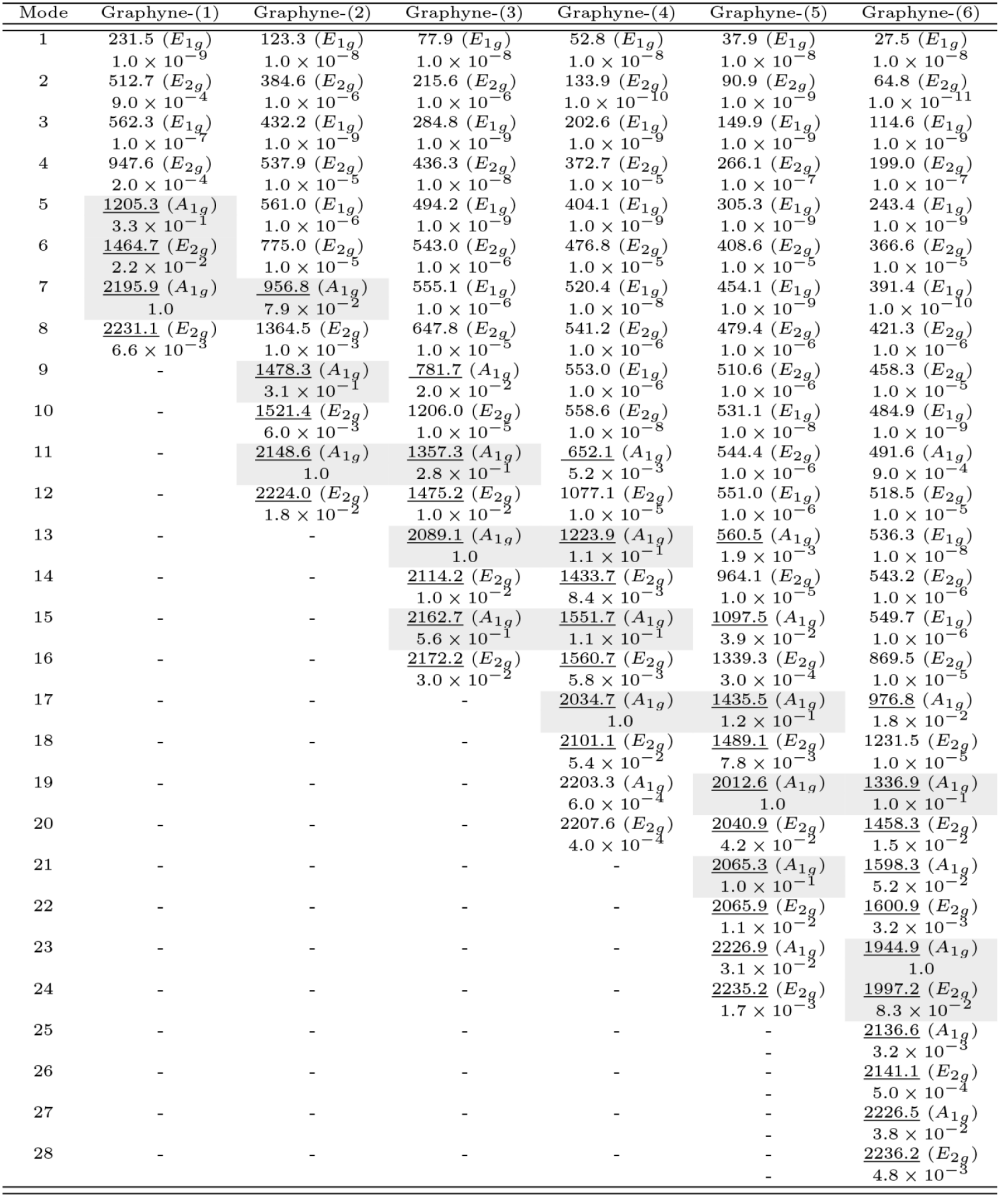
Raman-Active Phonons in Graphyne-(1 to 6)^a^

a Their wavenumbers (cm^–1^) and irreducible representations (in parentheses) are in the row of the mode number (labeled in increasing wavenumber order), and the scattering intensities (normalized by the highest value for each graphyne-(*N*)) are in the row below it. The underlined wavenumbers are for modes exhibited in Figure 3, and those shaded in gray are the three most intense Raman peaks.

Figure 3 can be divided into two wavenumber groups: the one spanning from 1600 cm^–1^ to 2400 cm^–1^ (see the long dash on the right) due to the movement of acetylene-like groups, characteristic of materials that display *sp* carbon chains;^63^ the other spanning in the adjacent lowest wavenumber region, due to the movement of the benzene-like units only, characteristic of materials with *sp* ^2^ carbons, and also due to the movement of both acetylene- and benzene-like units, characteristic of *sp*–*sp* ^2^ carbon materials.

The weaker features in the Raman spectra of graphyne-(1, 3, 4, 5, and 6) at 1464.7 cm^–1^, 1475.2 cm^–1^, 1433.7 cm^–1^, 1489.1 cm^–1^, and 1458.3 cm^–1^, respectively, can be viewed as the analogue of the G band in graphitic materials.^63–65^ First, these modes display *E*_2*g*_ symmetry, where the carbyne chains are essentially motionless, and the *sp* ^2^ carbons of the benzene-like units display an in-plane stretching pattern, e.g., the case for graphyne-(1) is shown in the inset in Figure 3. Second, their Raman shifts are close in wavenumber frequency to that of the graphene G band.

The most intense Raman peaks in graphyne-(1 to 6) are due to the *A*_1*g*_ modes at 2195.9, 2148.6, 2089.1, 2034.7, 2012.6, and 1944.9 cm^–1^, respectively. These modes compose the Y band,^31–33,41,42^ whose vibration patterns are illustrated in Figure 4.

**Figure 4:**
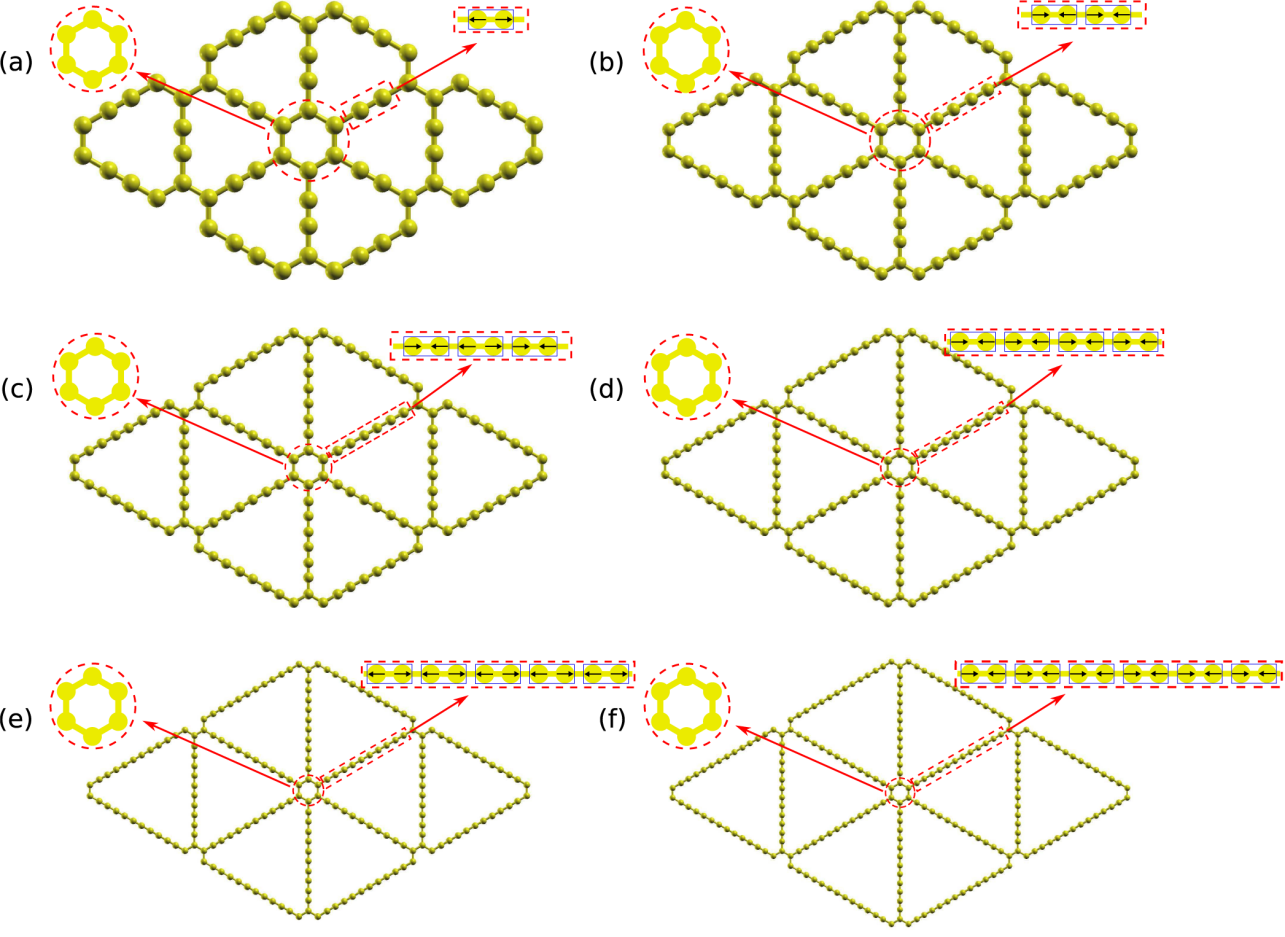
Vibration pattern of the carbyne chains with *A*_1*g*_ symmetry, which give rise to the highest Raman peaks in the Y band of (a) to (f) graphyne-(1 to 6), respectively.

The vibration patterns of the Y band are characterized by the in-plane vibration of the acetylene-like units, where adjacent carbon atoms vibrate out-of-phase, i.e., the triple bonds (−C≡C−) vibrate in-phase,^63^ while the benzene-like units are motionless.

Another characteristic pattern group in graphyne-(*N*) is the acetylene-like units vibrating with *E*_2*g*_ pattern symmetry, with no participation of the benzene-like units; the Y’ band,^63^ shown in Figure 5. The Raman shifts of the Y’ band in graphyne-(1 to 6) are 2221.1, 2224.0, 2172.2, 2101.1, 2040.9, and 1997.2 cm^–1^, respectively.

**Figure 5:**
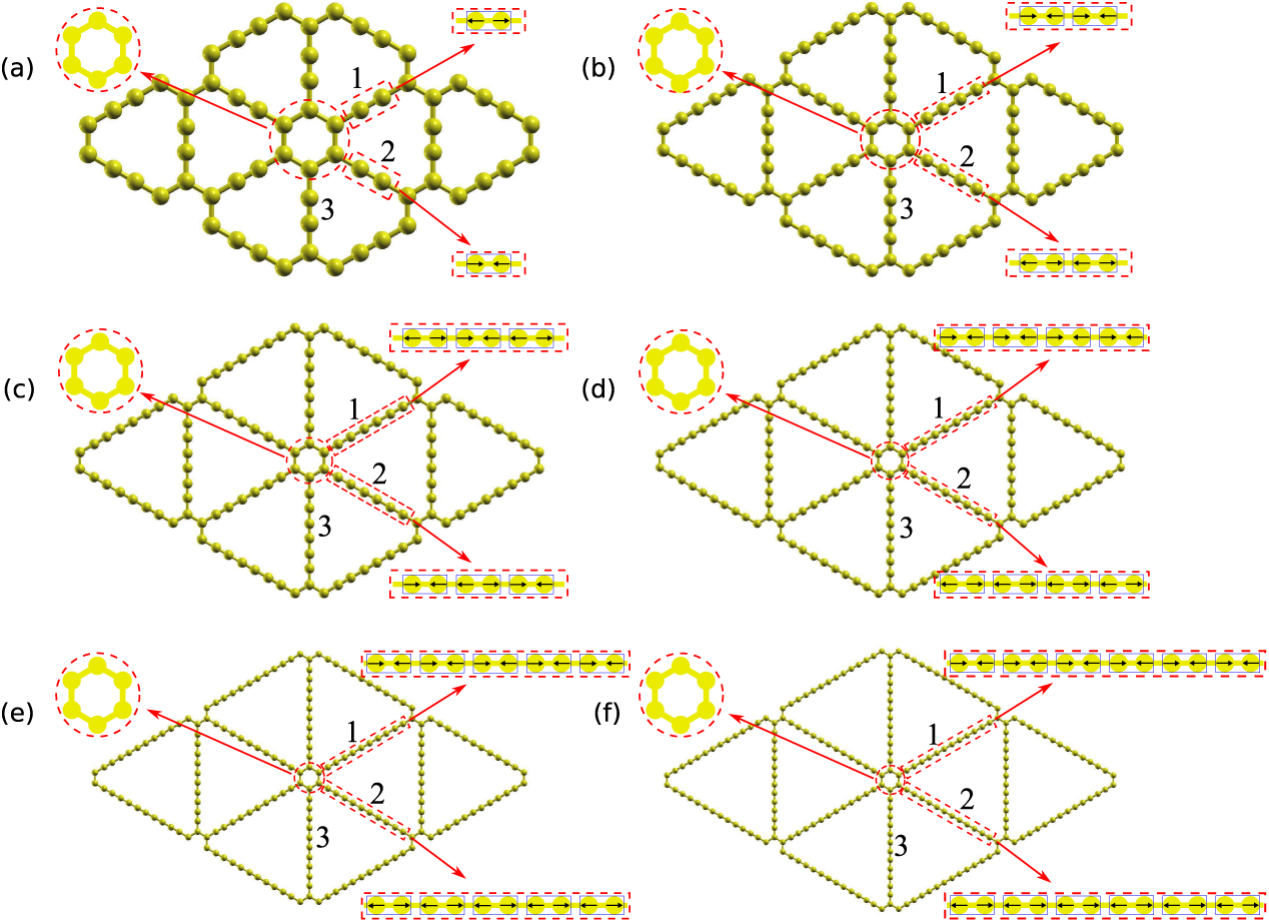
Vibration pattern graphyne-(*N*) carbyne chains with *E*_2*g*_ symmetry, which give rise to the Y’ band. (a–f) graphyne-(1 to 6), respectively. Some carbyne chains are labeled with the numbers 1, 2, and 3. Their opposite chains with respect to the carbon ring display the same vibration pattern.

In the Y band (Figure 4), all carbyne chains display the same pattern, resulting in the 1D totally symmetric *A*_1*g*_ mode, where the *D*_6*h*_ group symmetry is preserved. On the other hand, the band Y’ comes from the two-dimensional *E*_2*g*_ symmetry mode, where the atomic vibrations break the *D*_6*h*_ symmetry. Figure 5 shows the vibration pattern of one of the degenerate atomic displacements of the *E*_2*g*_ mode that give rise to the Y’ band, where the carbyne chains labeled 1 and 2 have out-of-phase patterns and that labeled 3 is motionless (and the carbyne chains opposite to these with respect to the carbon ring display the same vibration pattern).

Figure 6 presents the *N*-dependence of the Y band (*A*_1*g*_ modes; Figure 4) and the Y’ band (*E*_2*g*_ modes; Figure 5).

**Figure 6:**
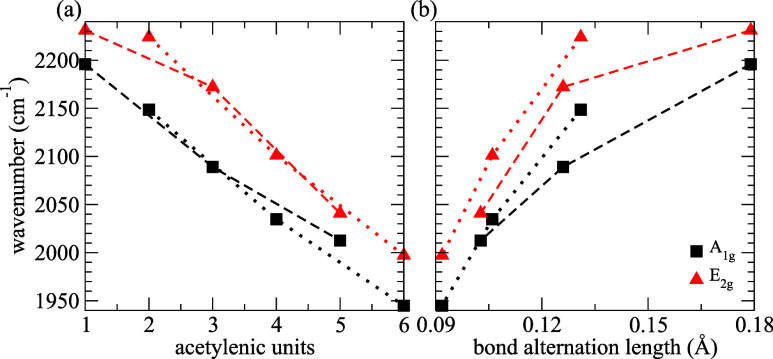
Dependence of the wavenumbers of the Y and Y’ bands in graphyne-(*N*) with respect to the (a) number of acetylenic units and the (b) bond alternation length. Dotted and dashed lines are guides for the eye of the *N*-even and *N*-odd cases, respectively. Squares and triangles represent the symmetries of the Y and Y’ bands, respectively.

Figure 6a shows a decrease in the Raman shifts of both Y and Y’ bands with the increase in *N*. In Figure 6b, we present the *N*-dependence of these bands with BLA. The results indicate that the Raman shifts of both Y and Y’ bands decrease with the decrease of the BLA (increase of the π-conjugation). In more detail, the Raman shifts of the structures with *N*-even and *N*-odd converge to the same trends when the BLA decreases. This softening of the Y and Y’ bands with the increase of *N* (increase of π-conjugation) that we found is also observed in carbon atom wire systems.^7^ In a simplified view, the frequency of vibrations in crystals is given by

4 $$\omega \propto \sqrt{\frac{k}{\mu }}$$

where *k* is the force constant associated with the atomic displacement pattern of the mode, also related to the crystal’s stiffness coefficients, and μ is the effective mass related to the atomic vibrational pattern of the mode.

Thus, the reduction of graphyne-(*N*) Young’s modulus with the increase of the carbyne chain^28,30^ may explain the trends of the results in Figure 6, as the stiffness of a material and the wavenumber of its vibrations increase (decrease) with increases (decreases) in the interatomic force constants. Another viewpoint to rationalize the trends in Figure 1a is that the triple bonds are stiffer than the single bonds in the carbyne chains of graphynes-(*N*),^30^ i.e., the vibration mode wavenumber is expected to be more sensitive to variations in the length of the triple bonds. Figure 2c shows that the length of triple bonds increases with the increase in *N*, reducing the interatomic force constants in these bonds, which in turn leads to the reduction in the Raman shift of the related vibration pattern, in agreement with the trends in Figure 6.

The G-like and G’-like vibrational modes in graphyne-(*N*) are presented in Figure 7.

**Figure 7:**
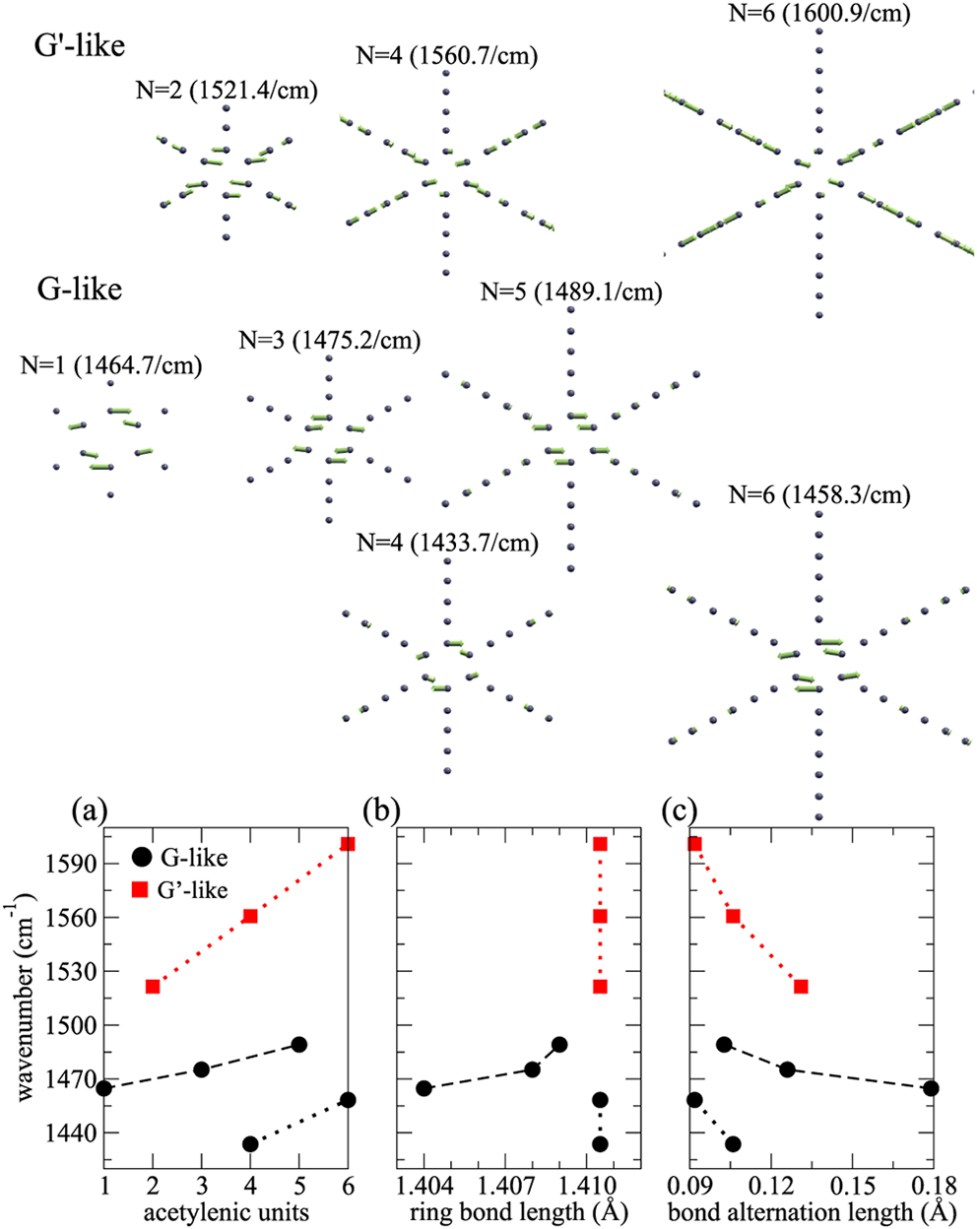
Atomistic models of the calculated G’-like and G-like vibrational patterns in graphyne-(*N*); the corresponding results for the wavenumbers are displayed in parentheses. G’-like (red, squares) and G-like (black, circles) wavenumber dependence with (a) the number of acetylenic units, (b) the bond length of the carbon ring, and (c) the bond alternation length. The results for *N*-even and *N*-odd are connected by dots and by dashes, respectively.

The mode where the CB0 stretches, similar to the graphene G band, and some carbons in the carbyne chain vibrate with a comparable magnitude, is present in the *N*-even graphyne-(*N*). This mode displays the *E*_2*g*_ symmetry, and we name it G’. The carbon ring stretching with apparent lack of vibration of the carbyne chain was called the G band, although in graphyne-(*N* ≥ 3) we notice the vibration of some carbons in the carbyne chain, yet with roughly 1 order of magnitude smaller displacements than those in the carbon ring. In addition, this mode is not present in graphyne-(2). We present the calculated atomic displacement patterns of the G-like and G’-like bands in Figure 7.

In Figure 7a–c, the wavenumbers of the G-like band are represented by black circles, and the results related to the *N*-even and *N*-odd structures are connected by dots and dashes, respectively. The G’-like band is represented by red squares. It only appears in the *N*-even structures, and we also connect them by using dots. The results in Figure 7a indicate that the G-like and G’-like band wavenumbers increase at different rates with *N*. In the case of the G-like band, we can distinguish between the trends for the *N*-even and *N*-odd cases. In Figure 7b, the shifts in the G-like band for *N*-even structures and G’-like band seem not correlated to the bond length of the carbon ring, which increases by a few mÅ in graphyne-(*N*-odd) and by a fraction of mÅ in graphyne-(*N*-even), respectively. The bond alternation length (BLA) seems to be a better geometrical parameter instead. In Figure 7c, we notice three different trends: for the G-like bands for *N*-even and *N*-odd structures, and for the G’-like band.

The experimental Raman spectra of graphyne-(2) and graphyne-(4) bulk samples reported in refs 31,32 display features near 1570 cm^–1^ and 1581 cm^–1^, respectively, characteristic of G-like bands, and features near 1926 cm^–1^ and 2190 cm^–1^ in graphyne-(2), and 2191 cm^–1^ in graphyne-(4), characteristic of the Y and Y’ bands. In those spectra, the Y and Y’ bands are barely observable, and the G-like band is quite broad, pointing to the large presence of sp^2^ amorphous carbon. For graphene and graphite, the G band is essentially thickness-independent.^14^ We also expect a negligible thickness dependence in the G-like, Y, and Y’ bands in graphynes-(*N*). Thus, our results for the monolayer are in qualitative agreement with the available experimental results for the bulk.

## Conclusions

We applied first-principles calculations to analyze the structural properties and the Raman spectrum of γ-graphyne with 1 to 6 acetylene-like groups in their carbyne chains. We found that the bond lengths in the carbon ring are equal by symmetry and essentially independent of the number *N* of acetylenic units, when *N* is even, and increase monotonically for *N*-odd structures, converging to the bond length of the structures with *N*-even. The BLA increases with the increase in the carbyne chain, characteristic of the increase in the π-conjugation in the carbyne chains. The unit cell length and nanopore area increase with *N* and *N* ^2^, respectively. The Raman shifts and vibrational symmetry patterns of these materials were calculated for *N* equal to 1 to 6, and the selection rules of the Raman-active mode were determined for an arbitrary *N*-value. The Y and Y’ modes, related to the carbyne chain stretching, soften with the increase of *N*, thus with the increase of π-conjugation. The G-like band, related to stretching of the benzene-like ring, and the G’-like band, related to both the stretching of the benzene-like ring and carbyne chains, increase with the decrease of the BLA, with different trends for the *N*-even and *N*-odd structures.

## Data Availability

The data supporting this article have been included in the main text. Additional raw data required to reproduce these findings can be made available upon reasonable request to the author.
